# Calorie restriction is the most reasonable anti-ageing intervention: a meta-analysis of survival curves

**DOI:** 10.1038/s41598-018-24146-z

**Published:** 2018-04-10

**Authors:** Yaru Liang, Chang Liu, Maoyang Lu, Qiongye Dong, Zimu Wang, Zhuoran Wang, Wenxiang Xiong, Nannan Zhang, Jiawei Zhou, Qingfei Liu, Xiaowo Wang, Zhao Wang

**Affiliations:** 1 MOE Key Laboratory of Protein Sciences, School of Pharmaceutical Sciences, Tsinghua University, Beijing, 100084 China; 2 MOE Key Laboratory of Bioinformatics and Bioinformatics Division, Center for Synthetic and System Biology, TNLIST/Department of Automation, Tsinghua University, Beijing, 100084 China; 3 School of Data Sciences, Zhejiang University of Finance & Economics, Zhejiang, 310018 China

## Abstract

Despite technological advances, the survival records from longevity experiments remain the most indispensable tool in ageing-related research. A variety of interventions, including medications, genetic manipulations and calorie restriction (CR), have been demonstrated to extend the lifespan of several species. Surprisingly, few systematic studies have investigated the differences among these anti-ageing strategies using survival data. Here, we conduct a comprehensive and comparative meta-analysis of numerous published studies on *Caenorhabditis elegans* and *Drosophila*. We found that CR and genetic manipulations are generally more effective than medications at extending the total lifespan in both models, and CR can improve the ageing pattern of *C*. *elegans*. We further analysed the survival variation for different anti-ageing medications and determined that hypoglycaemic agents and antioxidants are advantageous despite only moderately increasing the overall lifespan; therefore, these two types of medications are promising CR mimetics. Analysis of genetic manipulations also indicated that the genes or pathways that extend lifespan in a healthier pattern are associated with CR. These results suggest that CR or CR mimetics may be the most reasonable and potentially beneficial anti-ageing strategy.

## Introduction

Research on the biology of ageing has been conducted for centuries. Survival curves showing the surviving proportion of a population *versus* time are an intuitive means of illustrating the whole lifespan of a group of organisms and remain a key component of ageing research. Various anti-ageing interventions have been demonstrated to extend the lifespan of model organisms ranging from nematodes to fruit flies to rodents^1–4^, with contradictory reports in rhesus monkeys^5^. These interventions have mainly included calorie restriction (CR), genetic manipulations, and pharmaceutical administration^1,6^.

However, whether these interventions extend the lifespan via universal or distinct patterns remains unclear. Traditionally, in ageing research, survival data from lifespan experiments are mainly analysed in the original study, and data are not collected and stored together. Meta-analyses^7^ are mainly limited to either sufficiently large subsets of survival data acquired under identical conditions or the application of methods accounting for varying additional factors. The published meta-analyses of survival data have mostly assessed CR^8–10^. For example, reportedly, CR significantly extends lifespan, and the proportion of protein intake is more important for lifespan extension than the degree of CR^9^. No study has demonstrated whether CR, genetic manipulation or pharmaceutical administration is superior at extending lifespan and delaying ageing.

Here, we attempted to resolve this question by conducting a comprehensive and comparative meta-analysis of the effect patterns of these different interventions and their corresponding mechanisms via survival curves. We have focused our analyses on *Caenorhabditis elegans* and *Drosophila*, powerful model systems that are widely used in ageing research. We developed an algorithm that enabled us to combine multiple strains of these species from a large number of studies and to extract general trends from relevant results. Our main aims were as follows: (i) to investigate the effect patterns of different anti-ageing interventions on survival curves and to identify the most effective and healthiest interventions; (ii) to determine whether the effect on longevity is conserved between *C*. *elegans* and *Drosophila*; and (iii) to uncover the pattern of potential anti-ageing mechanisms between different interventions. Our re-analysis of survival data using this new method highlights the overall advantages of CR in delaying ageing and provides a direction for the discovery of effective anti-ageing strategies.

## Results

### Changes in the size and shape of survival curves represent different anti-ageing effect patterns

We obtained survival data from graphs^11,12^ extracted from the retrieved literature by using Gompertz model to fit the survival curve with the maximum-likelihood estimation (MLE)^13^. The Gompertz model describes the survival rate using the equation $S(t)={e}^{-\frac{a}{b}({e}^{bt}-1)}$. Finally, we identified 284 studies that fit our inclusion criteria, including 46 case-control pairs of survival curves of *C*. *elegans* and 238 pairs of *Drosophila* (Supplementary Data S1, Supplementary File S1).

**Figure 1:**
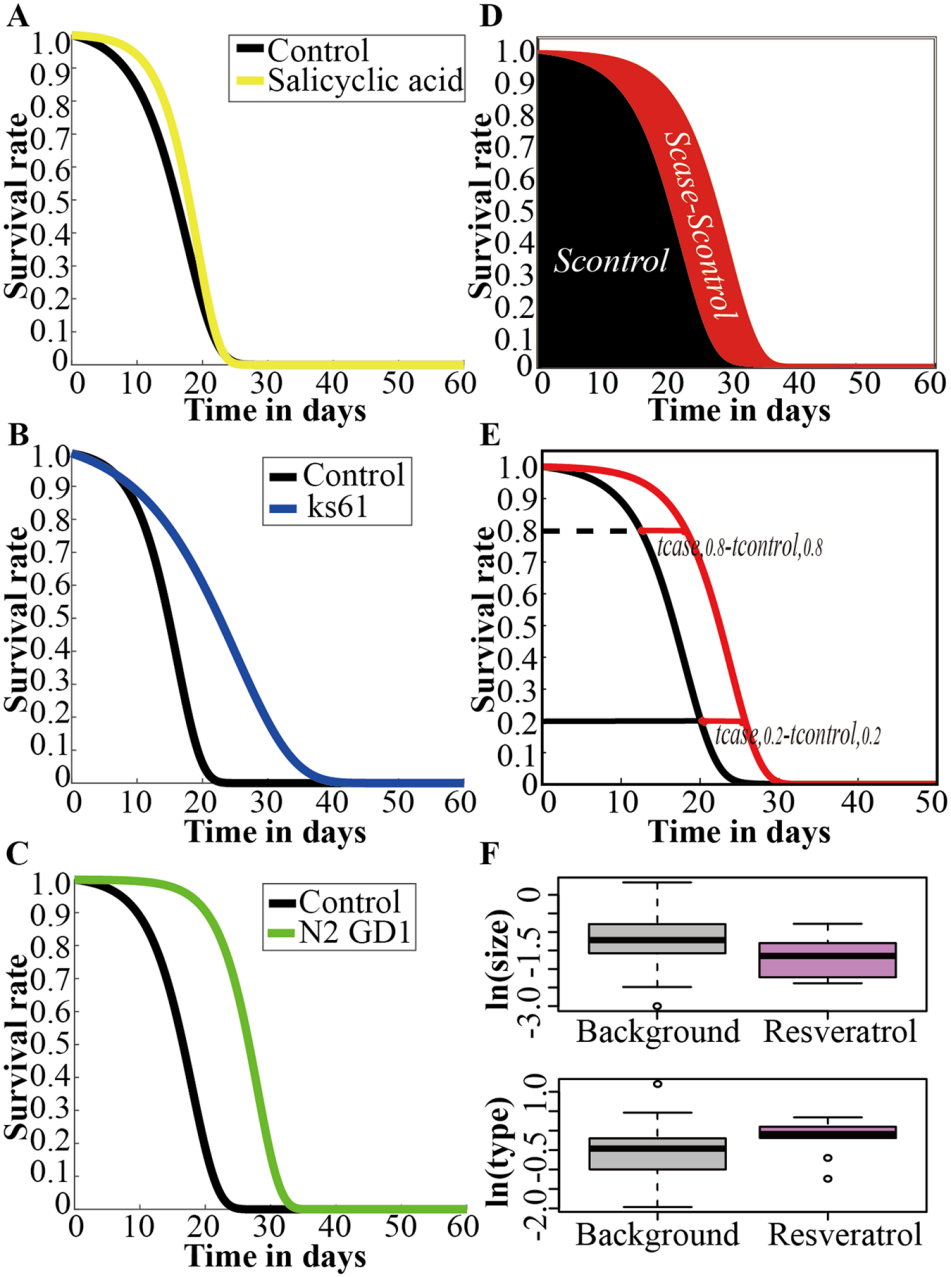
Schematic diagram of the cluster features. (**A**–**C**) Different anti-ageing effect patterns (e.g., salicylic acid: sharp improvement, ks61: slow improvement, N2 GD1: parallel improvement). (**D**) Size improvement. (**E**) Anti-ageing type features. (**F**) Boxplot of the visualized features of resveratrol and other treatments in *C*. *elegans* demonstrating that the survival curves generated using identical factors have similar features.

We found that different shift patterns exist among different anti-ageing interventions. For example, salicylic acid extends lifespan through a sharp improvement, as the extension occurs in the early stage (Fig. 1A). Conversely, ks61 extends lifespan through a slowly improvement, as most of the extension occurs in the late stage (Fig. 1B). In addition, N2 GD1 extends lifespan through a parallel pattern, as the improvement is the same in each stage of ageing (Fig. 1C). Therefore, we can infer that different anti-ageing interventions may extend lifespan in different ways or patterns.

Because the survival curves assessed here were collected from many published papers, environmental factors such as the temperature, medium, strain and sex were not uniform and varied significantly among different laboratories (Supplementary Fig. S1). We clustered survival curves by sampling certain survival points, and using the above factors to cluster the points. Fisher’s test was applied to calculate the enrichment of each cluster and condition above. These variations led to significant differences even between normal control samples (Supplementary Tables S1 and S2), hindering the direct comparison of the effects of different anti-ageing interventions. We therefore proposed a method to extract relative features and measure anti-ageing effects compared to those of the control, including the size improvement and anti-ageing type features (Fig. 1D and E). Importantly, survival curves for the same factor from different laboratories had similar feature distributions, thus further supporting the feasibility of our method (Fig. 1F, Supplementary File S2). We examined not only the degree of the total size improvement but also the pattern of delayed ageing. For a population, a pattern occurring mainly in early life stages could improve the population structure, whereas a pattern effect occurring later in life would likely present more of a burden to society. Therefore, the parallel shift pattern, with both a large ln(size) and an ln(type) close to 0, may be the ideal effect of the lifespan-extending method because it improves survival over the whole lifespan of the population and does not change the lifespan structure, preserving the stability of the population.

### CR and genetic manipulations are effective ways to extend total lifespan

In general, anti-ageing interventions in these two organisms are mainly classified into three categories: CR, medication administration, and genetic manipulations (Supplementary Data S1). The differences among these three types of anti-ageing interventions were extracted and expressed as features of the survival curves of each control and case cohort; we also compared the effects of different interventions on the survival curves from a biological perspective.

**Figure 2:**
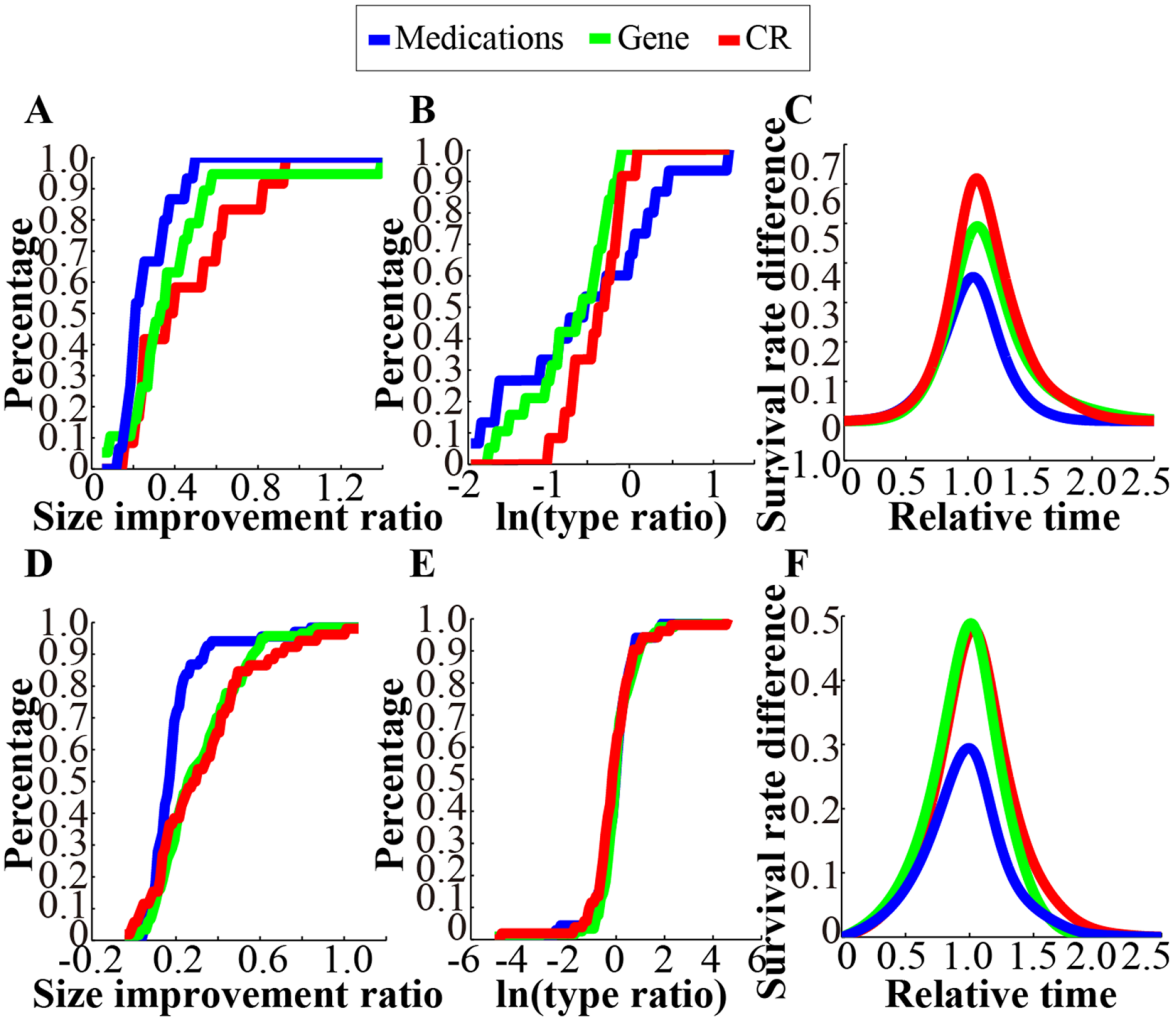
Analysis of different classes indicating that CR is more effective at changing the shape and scale of the survival curves. (**A**) Cumulative distribution of the size improvement in *C*. *elegans*. (**B**) Cumulative distribution of the type ratio in *C*. *elegans*. (**C**) Average case-control difference curves of *C*. *elegans*. (**D**) Cumulative distribution of the size improvement in *Drosophila*. (**E**) Cumulative distribution of the type ratio in *Drosophila*. (**F**) Average case-control difference curves of *Drosophila*.

The visualized feature scatter and corresponding cumulative distribution plot of *C*. *elegans* indicated that the degrees of the size improvement due to CR and genetic manipulations were slightly larger than that due to medications, even though the *P*-value was not statistically significant (Fig. 2A, Supplementary Table S3; Kolmogorov-Smirnov (KS) test: *P*_*CR-medications*_ = 0.09878, *P*_*gene-medications*_ = 0.06524). However, genetic manipulations mainly increased the maximum lifespan, whereas CR improved the total lifespan (Fig. 2B, Supplementary Table S3). As illustrated by the average difference curves (see the curve comparison in the Methods), the mode of improvement due to CR seemed to benefit types of individuals in the group, whereas genetic manipulations appeared to benefit only a few long-lived individuals. Thus, CR is more beneficial for a population because more individuals live longer, in contrast to genetic manipulations, which allows a few long-lived individuals to expend more resources to sustain life (Fig. 2C, Supplementary Fig. S2A). Similarly, the meta-analysis of *Drosophila* indicated a genetic transitivity (Fig. 2D and E, Supplementary Table S3; KS test: *P*_*CR-medications*_ = 1.476e-04, *P*_*gene-medications*_ = 2.132e-06), although the effect patterns of CR and genetic manipulations did not differ significantly (Fig. 2E, Supplementary Table S3), as evident from the average difference survival curves (Fig. 2F, Supplementary Fig. S2B). We therefore concluded that CR and genetic manipulations resulted in a large degree of improvement in lifespan compared to medications, although the underlying mechanism of this improvement is unknown. Nevertheless, the effect pattern of CR is superior to that of genetic manipulations in *C*. *elegans*.

### Hypoglycaemic agents and antioxidants improve survival throughout the entire lifespan

CR and genetic manipulations have greater effects on extending lifespan than the use of medications. However, genetic manipulation appears to have limited potential for direct application in humans^14–16^, and most people would not comply with such a rigorous CR programme because it may reduce quality of life^17–19^. As an alternative strategy, new research has focused on the development of ‘CR mimetics’, which are compounds that mimic CR by targeting the metabolic and stress pathways affected by CR but without actually restricting caloric intake^20–23^. For example, medications that inhibit glycolysis (2-deoxyglucose), enhance insulin action (metformin), or affect stress-signalling pathways (resveratrol), are being assessed as CR mimetics^24^. We therefore examined the lifespan effects of different classes of medications, which revealed some interesting patterns of differences.

**Figure 3:**
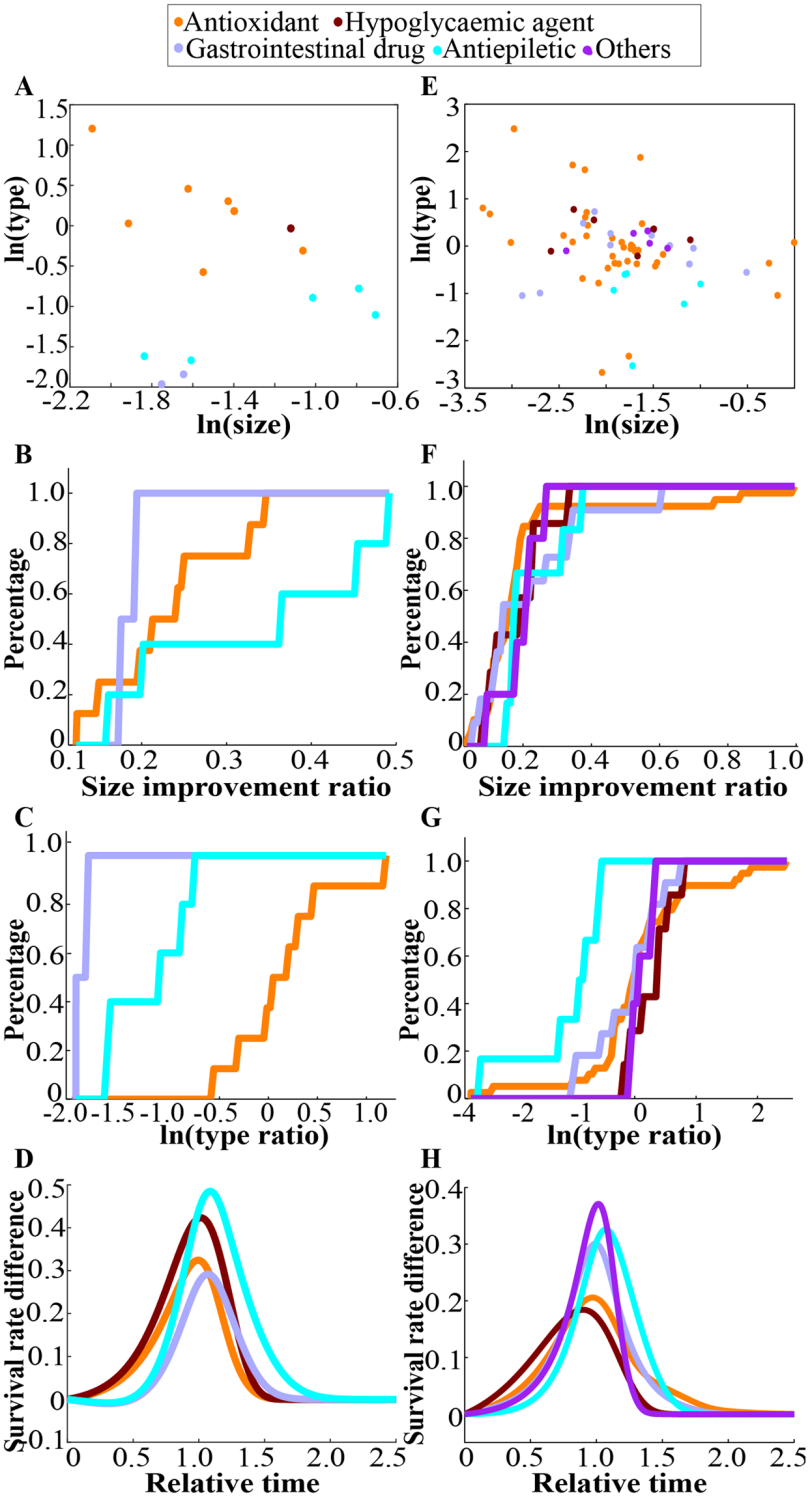
Analysis of different medications demonstrating that antioxidants and hyperglycaemic agents extend the lifespan of both models more effectively than other medications. (**A**) Visualized scatter distribution of different medications classified by the size improvement and type in *C*. *elegans*. (**B**) Cumulative distribution of the size improvements due to medications in *C*. *elegans*. (**C**) Cumulative distribution of the type features due to medications in *C*. *elegans*. (**D**) Average case-control difference curves of *C*. *elegans*. (**E**) Visualized scatter distribution of different medications classified by the size improvement and type in *Drosophila*. (**F**) Cumulative distribution of the size improvement due to medications in *Drosophila*. (**G**) Cumulative distribution of the type features due to medications in *Drosophila*. (**H**) Average case-control difference curves of *Drosophila*.

Medications were classified by their pharmacological action, clinical application or anti-ageing-related pathway (Supplementary Data S1). The KS test was applied to assess the pattern differences and the cumulative distribution of each feature, and a significant pattern was observed for classification by pharmacological action (Fig. 3A–C, Supplementary Table S4). The average survival curve differences indicated that the improvement due to antiepileptic medications was relatively large but mainly affected the later stage of life rather than the early stage and that other medications, including antioxidants and hypoglycaemic agents, can preserve the survival across the entire lifespan (Fig. 3D, Supplementary Fig. S3A, Supplementary Table S4; *P*_*type(Ao-Ae)*_ = 0.002928, *P*_*type(Ao-G)*_ = 0.04455), although the total improvement was not striking compared to that from other medication types. A cross-study comparison for *Drosophila* produced similar trends (Fig. 3E–H, Supplementary Fig. S3B, Supplementary Table S4; *P*_*type(Ao-Ae)*_ = 0.0003692, *P*_*type(Ao-G)*_ = 0.0458, *P*_*type(H-Ae)*_ = 0.001562). That is, though the total improvements due to hypoglycaemic agents and antioxidants were not as large as those due to antiepileptic medications, the former two types of medications shifted the survival curves in parallel, which might be a healthier way to extend lifespan of a population. Reportedly, the effects of hypoglycaemic agents and antioxidants on ageing, health, and lifespan are similar to those of CR^25,26^. Therefore, we can conclude that CR mimetics tend to be the most robust candidate among all the anti-ageing medications.

### Genetic manipulations that tend to extend lifespan in a healthier pattern are related to CR

**Figure 4:**
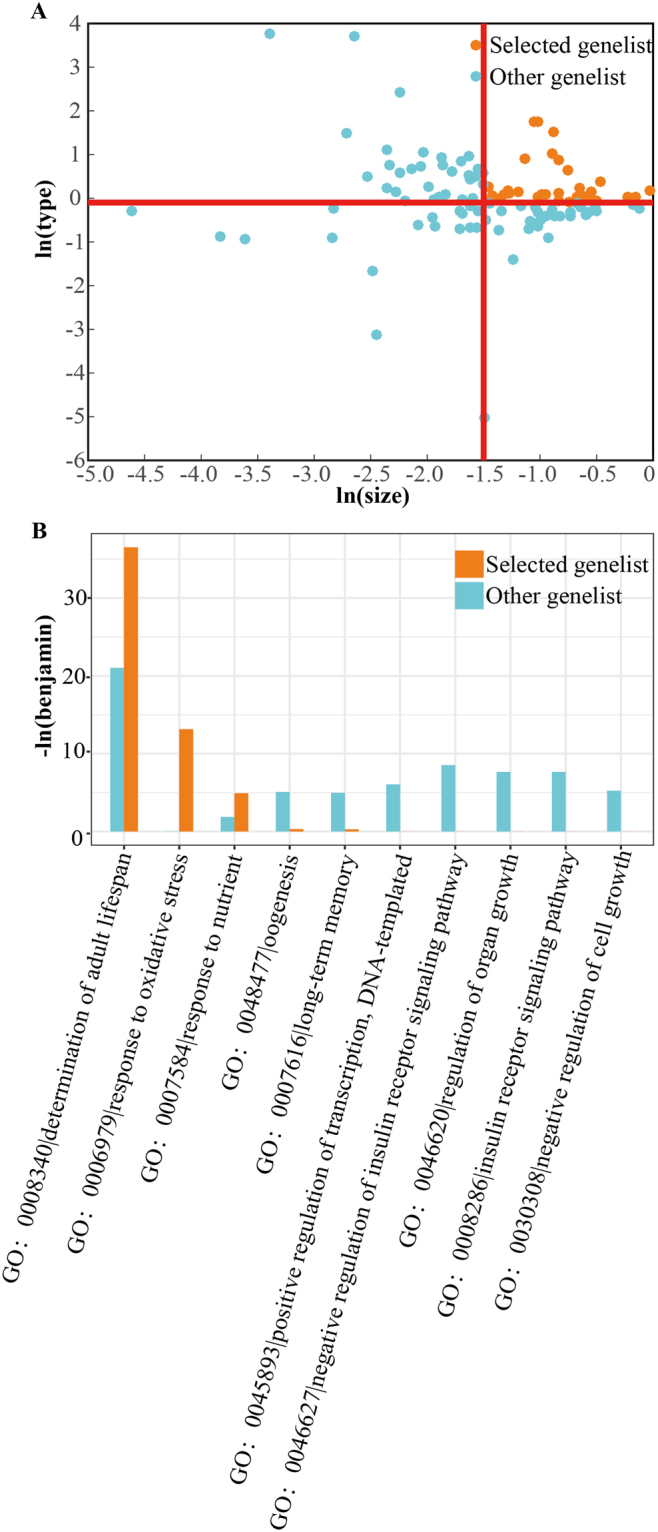
Analysis of genetic manipulations in *Drosophila* indicates that CR-associated genes extend lifespan in a better pattern than other analysed genes. (**A**) Visualized feature distribution of genes: selected genes (orange) and other genes (cyan). (**B**) Significantly enriched GO terms (only term ontologies with corrected *P*-values (Benjamin) ≤ 0.01 are shown).

Understanding the genetic basis of CR is of great importance not only to the biology of ageing but to the understanding of how diet can influence ageing, longevity, health and age-related diseases. Pharmaceutical interventions that target CR-associated genes are an emerging area with enormous potential. Therefore, we compared the biological functions of such genes in a selected region (a region with a larger size improvement and better anti-ageing pattern) with a background region (the other genes that we collected) and then analysed the biological pathway with GOTERM_BP_DIRECT in DAVID^27–29^ (Fig. 4A, Supplementary Data S2). The results showed that the enrichment pathways of the selected genes are largely associated with the determination of adult lifespan (GO: 0008340), oxidative stress (GO: 0006979) and nutrients (GO: 0007584) (Fig. 4B). The determination of adult lifespan category includes the JNK pathway, the insulin-signalling pathway, and the target of rapamycin (*dTOR*), which are important signalling pathways in CR^30^. In addition, the oxidative stress and nutritional response pathway is the basis for the lifespan-extending effect of CR^31,32^, reflecting the potential benefits of CR.

## Discussion

It is widely accepted that CR, medications and genetic manipulations extend lifespan in a diversity of species ranging from yeast to primates. However, no study has focused on the general differences and effect patterns of these interventions in lifespan experiments. For example, a recent study of the effects of a variety of lifespan interventions on *C*. *elegans* developed a good algorithm with an accelerated failure time (AFT) model to remove differences in the timescale to the same extent, but the question remained unresolved^33^. Here, we introduced a useful methodology that enables the variations in the scale and shape of survival curves to be measured separately, and we performed three procedures to reanalyse survival curves in two classic model organisms. First, we extracted relative features to measure the effects of distinct interventions, despite variations in the controls. We combined statistical analyses to validate the reliability of the results. Finally, we calculated the *P-*value of the KS test of each feature among different subtypes of anti-ageing interventions from a biological perspective.

**Figure 5:**
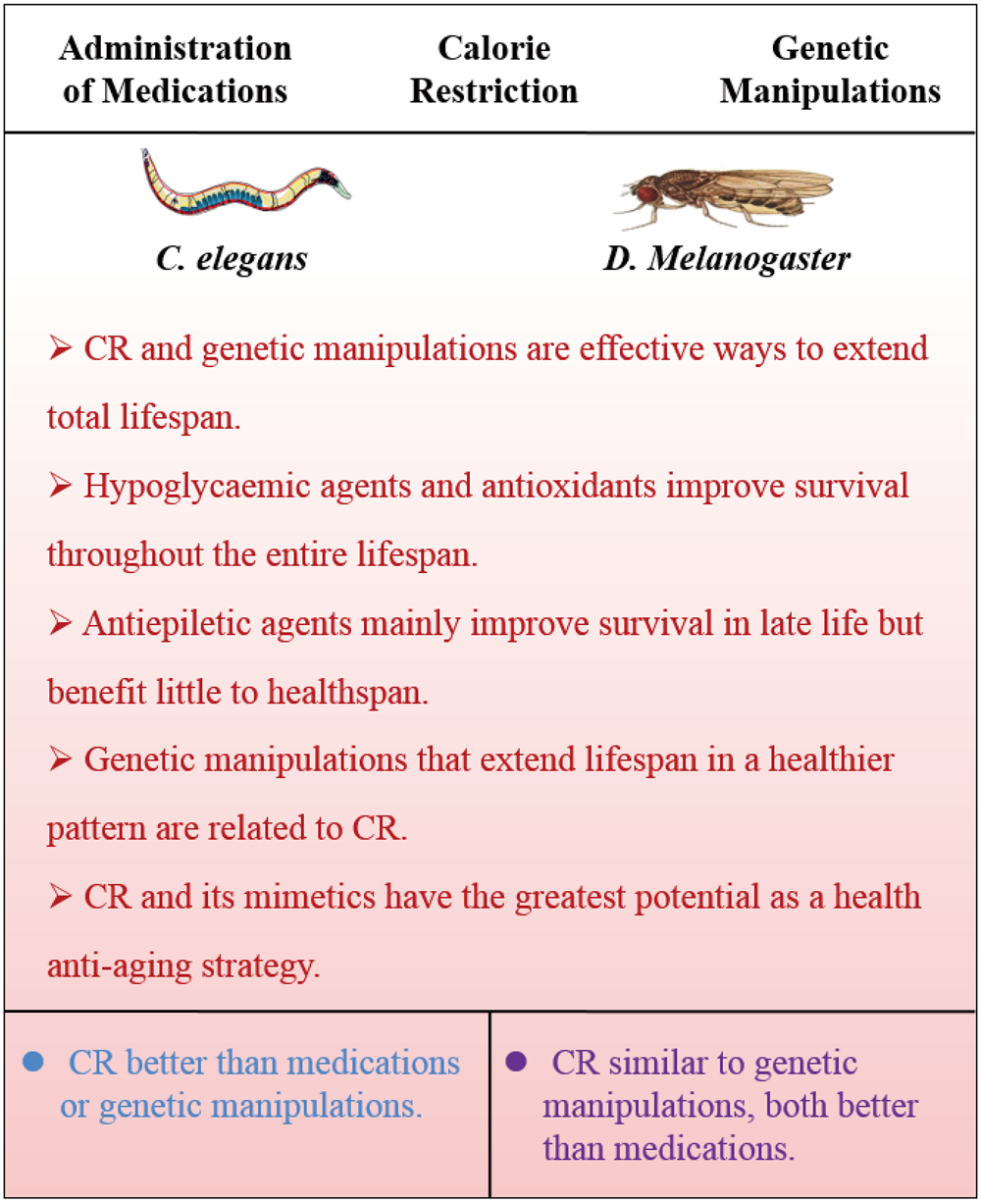
Graphical summary of the main results of our study. The effects of different anti-ageing interventions exhibited fairly strong species transitivity from *C*. *elegans* to *Drosophila*.

Our study indicated that CR and genetic manipulations are effective ways in delaying senescence. The effect pattern of CR is superior to that of genetic manipulation in *C*. *elegans* but similar to that of genetic manipulation in *Drosophila* (Fig. 5). Genetic manipulation in mammals faces many problems and risks, and CR, including changes in diet composition, time-restricted feeding or CR mimetics, could be a more feasible approach for humans. These considerations and our results support CR as a feasible and effective anti-ageing intervention.

Ageing is an inevitable aspect of life. Although the mechanism of ageing has not been fully elucidated, there are several general hypotheses for the ageing mechanism, including the free radical theory, decreased immune function, the telomere theory, and the brain ageing center doctrine^34–36^. Our research suggests that hypoglycaemic agents and antioxidants can obviously preserve the age structure of a population and delay senescence. These medications protect the cell membrane and organelles from free radical damage and can mimic the effects of CR. In summary, antioxidants and CR mimetics are key regulators for extending the lifespan of *C*. *elegans* and *Drosophila*.

One limitation of our study is the difficulty in obtaining unbiased data. We collected data from recently published papers and cannot control the bias of publishing. However, as most published papers have focused on the amount of the improvement (e.g., Log-rank test), our analysis results for the shape pattern may be more persuasive. Additionally, because original survival data were usually not supplied in the collected published papers, we could recover the data only using the Matlab ‘*digitize 2*’ package^37,38^ and could not apply the traditional Log-rank test or Cox hazard regression to analyse the differences in survival. Hence, we constructed a new method based on data recovery and model fitting. In addition, a SurvCurv database and online analysis platform for animal survival data was created, and all the survival records from the database originate from 60 publications^39–41^. Therefore, we checked all these publications to ensure that all the relevant survival data were included in our analysis. We used numerical survival data for *C*. *elegans* and *Drosophila* and specific analysis scripts to address these questions, which were otherwise very difficult to approach. Our research is currently based on a limited number of studies. The extension of these results to other organisms, such as mice or rats, requires a separate examination in future studies.

## Methods

### Data collection

Survival data from lifespan experiments were extracted from the literature. We searched both PubMed and Google Scholar using the keywords ‘calorie/dietary restriction/medications/gene’ + ‘longevity/lifespan’ + ‘*C*. *elegans*/*Drosophila*’ and examined the reference lists of the retrieved papers and reviews. Papers published on any date up to 2017 were included. From the more than 2000 studies that this search yielded, papers were selected that contained a graphical survival curve or, for some older studies, provided the complete data set from which a survival curve could be constructed. Our inclusion criteria were as follows: (i) Studies were conducted with the original empirical data using real animals and were not reviews or computer simulations. (ii) The experiment contained both a control group and a treatment group. (iii) Survival was reported until all animals died and was extractable from figures or tables in at least five binned time intervals. (iv) Only strains of control groups that were wild type were included in our analysis. (v) When multiple experimental groups (e.g., different medication concentrations) were available within the same study, we selected the experimental group for which the experimental protocol was most comparable to that of the control group. Accordingly, the following exclusion criteria were also used: (i) Strains of control groups that were transgenic or mutants were excluded. (ii) We were careful not to include multiple publications of the same data set, for example, the data set of the National Center for Toxicological Research (NCTR)/National Institute of Aging (NIA) cohorts^42^, which could lead to multiple inclusions of the same data, thereby biasing the meta-analysis. The Matlab ‘*digitize 2*’ package was used to recover survival data from figures.

### Model fitting for the survival curves

The survival curves in the various papers were generated from different numbers of samples and time intervals, preventing a direct comparison of the raw curves. Thus, we used parametric models to fit the raw data to smoothed curves. Six common mortality models, including the exponential model, logistic model, logistic-Makeham model, Weibull model, Gompertz model and Gompertz-Makeham model^13^, were applied to fit each cohort. The relative goodness of fit was measured by the Akaike^43,44^ and Bayesian^45^ information criterion (AIC and BIC, respectively) values and the classic MLE together with the parameter values provided. In general, the Gompertz model yielded good fit results for both the *C*. *elegans* and *Drosophila* survival data (Supplementary Data S1) and the Gompertz parameters provided a meaningful explanation^46^. This model was therefore used for the analyses.

### Feature selection

Because many factors affect survival, considerable variation was observed even between the survival curves for the normal control samples from the different papers, hindering a direct comparison of the curves for different treatments from different papers. We therefore extracted the features that measured the relative effects of each interaction compared to the matched control samples reported in the same paper. We predicted that a population begins to age when the survival rate decreases to 80%, and the corresponding time was represented as *t*_*survival*, *0*.*8*_. When the survival rate decreased to 20%, few individuals were still alive, and the corresponding time was represented as *t*_*survival*, *0*.*2*_. Therefore, the transition time was given by *t*_*transition*_ = *t*_*survival*, *0*.*2*_ *− t*_*survival*, *0*.*8*_. The survival curve for the transition time was approximated to be linear, and the features were extracted on this basis. The first feature was size improvement, which was used to measure the overall degree of improvement in lifespan and was defined as (*S*_*case*_*−S*_*control*_)/*S*_*control*_ (Fig. 1D). The second feature was the anti-ageing type, which was defined as Δ = (*t*_*case*,*0*.*8*_−*t*_*control*,*0*.*8*_)/(*t*_*case*,*0*.*2*_−*t*_*control*,*0*.*2*_). If Δ > 1, the shape of the curve change was an inverted trapezium; if Δ = 1, the curve shifted in parallel; and if Δ < 1, the shape of the curve change was a trapezium (Fig. 1E).

### Curve comparison method

We used case-control plots of *C*. *elegans* and *Drosophila* to study the differences between the case and control survival curves. To compare the shape and scale changes, the timeline was normalized by dividing by the control time at which the survival rate decreased to 20% on the same scale, and the size was reshaped by the improvement size. This method allowed us to compare different anti-ageing strategies using the transformed difference survival curves on the same plot.

### Gene set enrichment and gene ontology (GO) analysis

As those lifespan strategies that have a longer extension and a parallel pattern seem to be better strategies, we analysed the biological functions of the genes in the regions affected by such strategies by GO analysis using DAVID^27–29^. From this analysis, we can determine the function of these genes and can postulate whether the beneficial effects are associated with CR. We analysed the biological pathways and compared the results among these better regions and other genes that we collected.

### Significance analysis

We used two-sample KS tests to determine if two subgroups had different anti-ageing patterns and applied this test to the different features studied^47,48^. *P*-values were calculated from the KS statistic and were determined to be more or less significant using a one-sided test. We also used the KS test to determine which of the parameters in the Gompertz model produced significant improvements in survival as we tested the differences in the parameters between each control and case cohort.

## Electronic supplementary material

Supplementary Information

Supplementary Data S1

Supplementary Data S2
