# Supplementary material for: Calorie restriction is the most reasonable anti-ageing intervention: a meta-analysis of survival curves
Source: Sci Rep. 2018 Apr 10;8:5779. doi: 10.1038/s41598-018-24146-z (PMC5893623; doi:10.1038/s41598-018-24146-z)
Supplement: Supplementary file 1 — Supplementary Information [file 41598_2018_24146_MOESM1_ESM.doc]

**Calorie restriction is the most reasonable anti-ageing intervention: a meta-analysis of survival curves**

Yaru Liang^1^,*, Chang Liu^2^,*, Maoyang Lu^1^, Qiongye Dong^2^, Zimu Wang^3^, Zhuoran Wang^1^, Wenxiang Xiong^1^, Nannan Zhang^1^, Jiawei Zhou^1^, Qingfei Liu^1^, Xiaowo Wang^2, #^, and Zhao Wang^1, #^

^1^MOE Key Laboratory of Protein Sciences, School of Pharmaceutical Sciences, Tsinghua University, Beijing 100084, China

^2^MOE Key Laboratory of Bioinformatics and Bioinformatics Division, Center for Synthetic and System Biology, TNLIST/Department of Automation, Tsinghua University, Beijing 100084, China

^3^School of Data Sciences, Zhejiang University of Finance & Economics, Zhejiang 310018, China

*These authors contributed equally to this work

**^#^Corresponding author:**

Dr. Zhao Wang, School of Pharmaceutical Sciences, Tsinghua University, Beijing 100084, China.

Tel: + 86 10 62772240

Fax: + 86 10 62772675

E-mail: zwang@tsinghua.edu.cn

Dr. Xiaowo Wang, Department of Automation, Tsinghua University, Beijing 100084, China.

Tel: +86 10 62794294

Fax: +86 10 62783552

E-mail: xwwang@tsinghua.edu.cn

**Supplementary information**

**
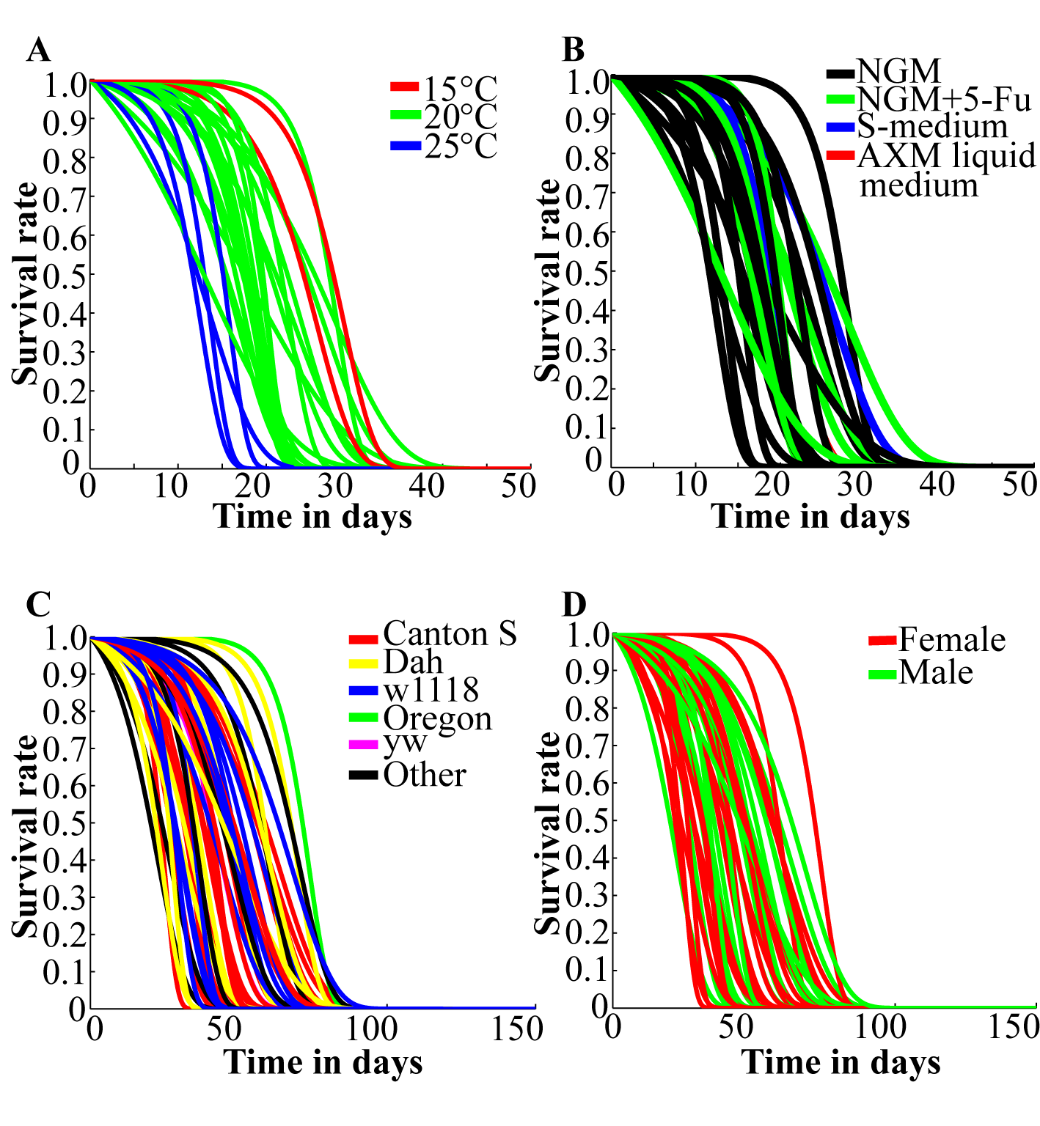
**

**Supplementary Figure S1. Significance analysis of normal survival curves of *C. elegans* and *Drosophila*.** (**A, B**) Survival curves of *C. elegans* classified by the temperature and medium. (**C, D**) Survival curves of *Drosophila* classified by the strain and sex.


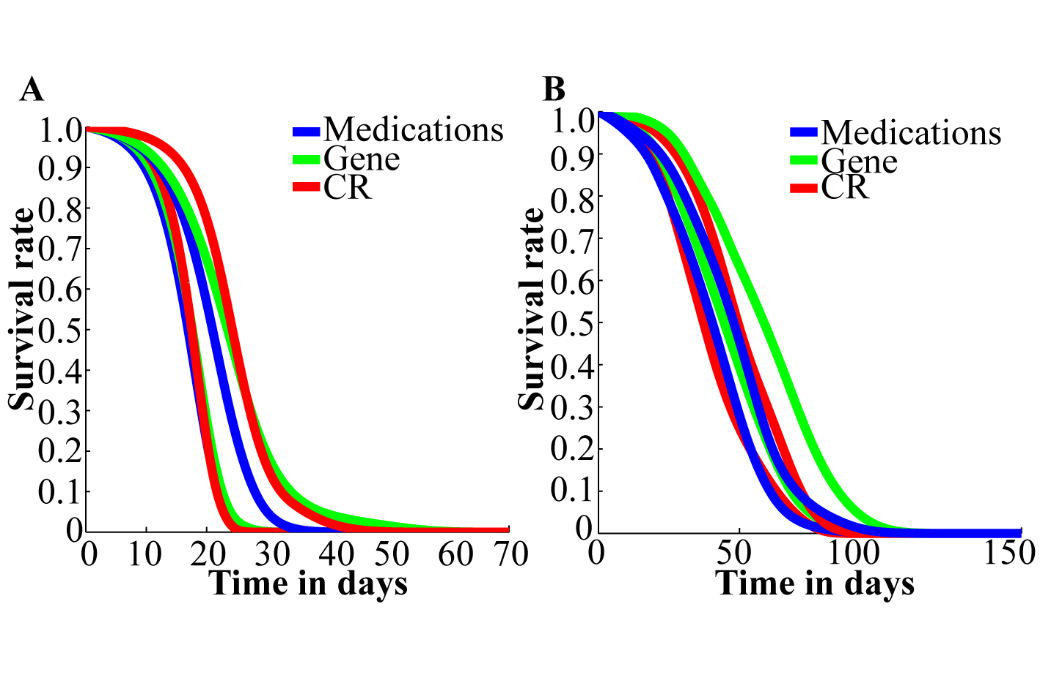


**Supplementary Figure S2. Average differences in the survival curves following three different anti-ageing interventions.** (**A**) *C. elegans*. (**B**) *Drosophila*.


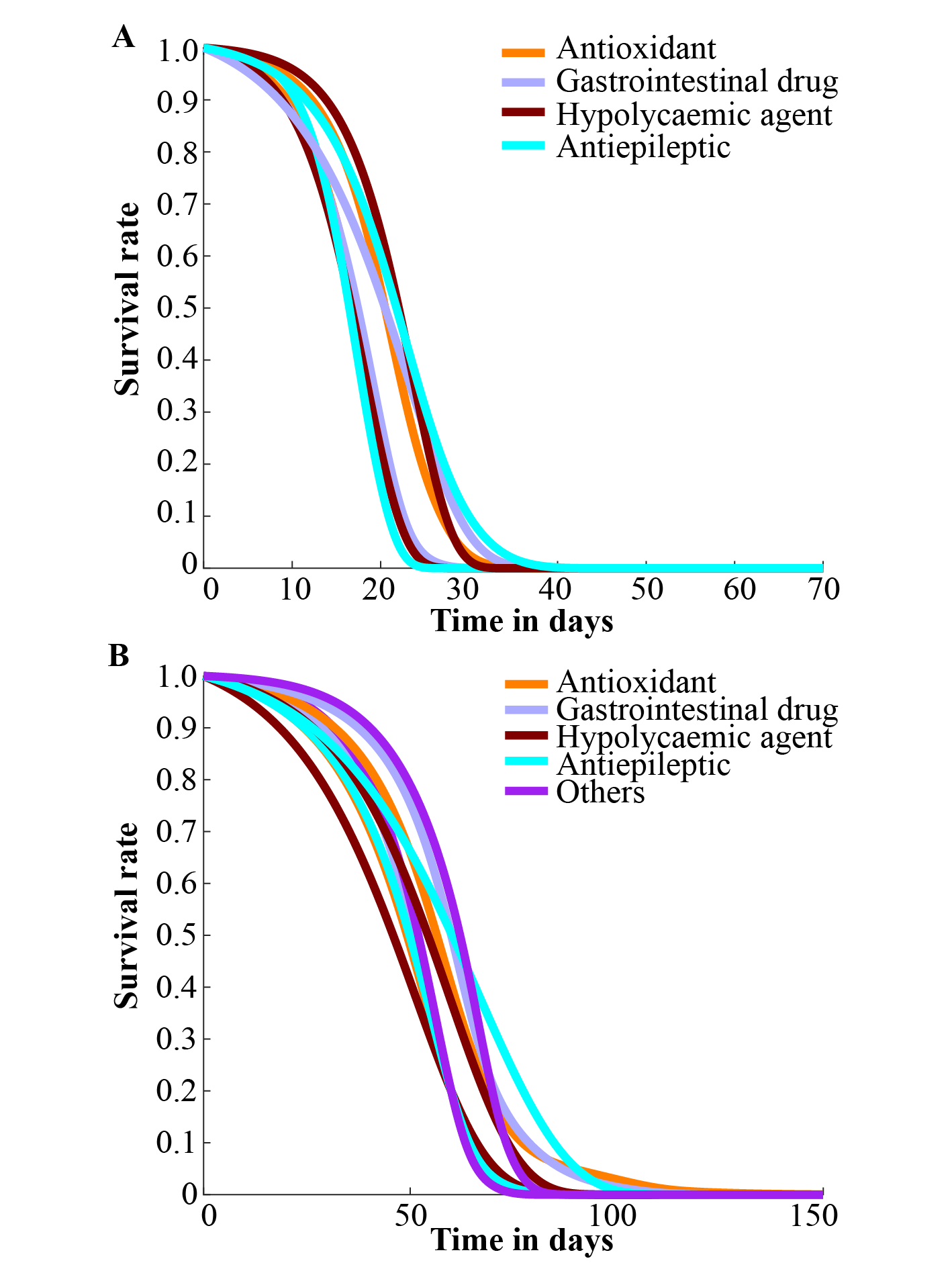


**Supplementary Figure S3. Average differences in survival curves following different anti-ageing medications.** (**A**) *C. elegans*. (**B**) *Drosophila*.

**Supplementary Table S1. Fisher’s test of two different classification results in *C. elegans*.**

| **Temperature/Medium** | **Cluster 1** | **Cluster 2** | **Cluster 3** | **Cluster 4** |
| --- | --- | --- | --- | --- |
| **15 ^o^C** | 1.00000 | 0.18010 | 1.00000 | 0.53080 |
| **20 ^o^C** | 0.99000 | 0.72720 | 0.03047 | 0.77960 |
| **25 ^o^C** | 0.01904 | 1.00000 | 0.99007 | 0.58120 |
| **NGM** | 0.08287 | 0.69380 | 0.47460 | 0.98069 |
| **NGM+5-Fu** | 0.89037 | 0.43540 | 0.67660 | 0.34982 |
| **S-Medium** | 1.00000 | 1.00000 | 0.48300 | 0.36946 |
| **AXM liquid medium** | 1.00000 | 1.00000 | 1.00000 | 0.09425 |

The *P-*values are from the meta-analysis using the Fisher’s exact test approach.

**Supplementary Table S2. Multi-factor analysis results in *Drosophila***.

| **Base** | **Cluster 1** | | **Cluster 2** | | **Cluster 3** | | | **Cluster 4** | |
| --- | --- | --- | --- | --- | --- | --- | --- | --- | --- |
| **1xSY** | 0.19641 | | 0.70239 | | 0.66664 | | | 0.27549 | |
| **normal SY/X** | 0.56979 | | 0.48400 | | 0.19916 | | | 0.81344 | |
| **Strain for Female** | **Cluster 1** | **Cluster 2** | | **Cluster 3** | **Cluster 4** | | **Cluster 5** | | **Cluster 6** |
| **Canton-S** | 1.0000 | 0.9329 | | 1.0000 | 1.0000 | | 1.0000 | | 1.0000 |
| **Dah** | 0.9162 | 1.0000 | | 1.0000 | 0.8985 | | 1.0000 | | 0.9328 |
| **W1118** | 1.0000 | 0.8985 | | 0.8985 | 1.0000 | | 0.8985 | | 1.0000 |
| **Oregon** | 1.0000 | 1.0000 | | 0.9700 | 1.0000 | | 1.0000 | | 1.0000 |
| **yw** | 1.0000 | 1.0000 | | 1.0000 | 1.0000 | | 1.0000 | | 0.8985 |
| **Other** | 0.9162 | 1.0000 | | 1.0000 | 1.0000 | | 1.0000 | | 1.0000 |
| **Strain for Male** | **Cluster 1** | **Cluster 2** | | **Cluster 3** | **Cluster 4** | | **Cluster 5** | | **Cluster 6** |
| **Canton-S** | 1.0000 | 1.0000 | | 1.0000 | 0.03833 | | 1.0000 | | 1.0000 |
| **Dah** | 1.0000 | 1.0000 | | 1.0000 | 1.0000 | | 1.0000 | | 1.0000 |
| **W1118** | 1.0000 | 0.8700 | | 0.04249 | 1.0000 | | 1.0000 | | 1.0000 |
| **Oregon** | 1.0000 | 1.0000 | | 1.0000 | 0.71209 | | 1.0000 | | 0.6553 |
| **yw** | 0.06681 | 0.7121 | | 0.71209 | 1.0000 | | 1.0000 | | 1.0000 |
| **Other** | 1.0000 | 1.0000 | | 1.0000 | 1.0000 | | 2.76e-5 | | 1.0000 |
| **Sex** | **Cluster 1** | | | | | **Cluster 2** | | | |
| **Female** | 0.7837365 | | | | | 0.01473721 | | | |
| **Male** | 0.02191579 | | | | | 0.57786434 | | | |

The *P*-values are from the meta-analysis using Fisher’s exact test approach.

**Supplementary Table S3. KS test of the significance of the visualized feature differences among medications, genetic manipulations and CR.**

| **Size improvement** | **CR-medications** | **Gene-medications** | **Gene-CR** |
| --- | --- | --- | --- |
| ***C. elegans*** | 0.09878 | 0.06524 | 0.7071 |
| ***Drosophila*** | 1.476e-04 | 2.132e-06 | 0.4542 |
| **Type** | **CR-medications** | **Gene-medications** | **Gene-CR** |
| ***C. elegans*** | 0.2273 | 0.6461 | 0.1868 |
| ***Drosophila*** | 0.2378 | 0.5493 | 0.1039 |

**Supplementary Table S4. KS test of the significance of visualized feature differences among anti-age**ing medications.

| ***C. elegans*** | | **Ao-Ae** | | **Ao-G** | | | **Ae-G** | | |
| --- | --- | --- | --- | --- | --- | --- | --- | --- | --- |
| **Size improvement** | | 0.1225 | | 0.2045 | | | 0.1606 | | |
| **Type** | | 0.002928 | | 0.04455 | | | 0.05743 | | |
| ***Drosophila*** | **Ao-H** | | **Ao-G** | | **Ao-Ae** | **Ao-O** | | **G-H** | |
| **Size improvement** | 0.2522 | | 0.2118 | | 0.1091 | 0.1167 | | 0.659 |  |
| **Type** | 0.1786 | | 0.0458 | | 0.0003692 | 0.225 | | 0.2729 |  |
| ***Drosophila*** | **H-Ae** | | **H-O** | | **Ae-G** | **G-O** | | **Ae-O** | |
| **Size improvement** | 0.3052 | | 0.7373 | | 0.09925 | 0.4029 | | 0.3049 |  |
| **Type** | 0.001562 | | 0.1489 | | 0.005528 | 0.4029 | | 0.004277 |  |

Ao: Antioxidants; Ae: Antiepileptic; G: Gastrointestinal; H: Hypoglycemic agents; O: Others.

**Supplementary Data S1. Summarized survival data that matched our selection criteria and classification results from a biological perspective.** Anti-ageing interventions were classified into medications, genetic manipulations and calorie restriction. The medications were further classified by their pharmacological effect, clinical application, medicinal property or signalling pathway.

**Supplementary Data S2. GO analysis.** Functional annotation results from the DAVID gene set enrichment analysis of genes that involved in the collected survival data. For each GO term, the selected and other gene numbers along with the statistical significance (-ln(Benjamin)) of the enrichment are presented.

**Supplementary File S1. References of articles from which the survival curves were extracted for the analysis of the lifespan extension pattern of different anti-ageing interventions (corresponding to the literature number in Supplementary Data S1, 1-64: *C. elegans*; 65-161: *Drosophila*).**

1. Wang, L. P., Jin, X., Huang, L., Li, P. F. & Wang, L. C. Mechanism of DhHP-6 on extending lifespan in *Caenorhabditis elegans*. *Journal of Jilin University* **50,** 1045-1048 (2012).
2. Ayyadevara, S., *et al*. Aspirin Inhibits oxidant stress, reduces age-associated functional declines, and extends lifespan of *Caenorhabditis elegans*. *Antioxid. Redox. Signal.* **18,** 481- 490 (2013).
3. Hunt, P. R., *et al*. Extension of lifespan of *C. elegans* by naphthoquinones that act through stress hormesis mechanisms. *PLoS One* **6,** e21922; 10.1371/journal.pone.0021922 (2011).
4. Wang, C. L., *et al*. Isolation, purification of extracellular polysaccharide from Viili and its effect on the lifespan of *Caenorhabditis elegans*. *Amino Acids Biotic Resources* **33,** 64-67 (2011).
5. Honda, Y., *et al*. Lifespan-extending effects of Royal Jelly and its related substances on the nematode *Caenorhabditis elegans*. *PLoS One* **6,** e23527; 10.1371/journal.pone.0023527 (2011).
6. Yang, W. X., *et al*. Retarding ageing effect of Toona Sinensis Leaf Flavonooids on *Caenorhabditis elegans*. *Modern Food Science and Technology* **26,** 931-933 (2010).
7. Han, H. J., *et al*. Study on the mechanism of quercetin on extending lifespan of *Caenorhabditis elegans*. *Amino Acids Biotic Resources* **33,** 35-38 (2011).
8. Onken, B. & Driscoll, M. Metformin induces a dietary restriction-like state and the oxidative stress response to extend *C. elegans* healthspan via AMPK, LKB1, and SKN-1. *PLoS One* **5,** e8759; 10.1371/journal.pone.0008758 (2010).
9. Evason, K., Collins, J. J., Huang, C., Hughes, S. & Kornfeld, K. Valproic acid extends *Caenorhabditis elegans* lifespan. *Aging Cell* **7,** 305-317 (2008).
10. Kim, J., *et al*. Effects of a potent antioxidant, platinum nanoparticle, on the lifespan of *Caenorhabditis elegans*. *Mech. Ageing Dev.* **129,** 322-331 (2008).
11. Petrascheck, M., Ye, X. L. & Buck, L. B. An antidepressant that extends lifespan in adult *Caenorhabditis elegans*. *Nature* **450,** 553-556 (2007).
12. Broué, F., Liere, P., Kenyon, C. & Baulieu, E. E. A steroid hormone that extends the lifespan of *Caenorhabditis elegans*. *Aging Cell* **6,** 87-94 (2007).
13. Evason, K., Huang, C., Yamben, I., Covey, D. F. & Kornfeld, K. Anticonvulsant medications extend worm life-span. *Science* **307,** 258-262 (2005).
14. Adachi, H. & Ishii, N. Effects of tocotrienols on life span and protein carbonylation in *Caenorhabditis elegans*. *J. Gerontol. A Biol. Sci. Med. Sci.* **55,** B280-285 (2000).
15. Greer, E. L. & Brunet, A. Different dietary restriction regimens extend lifespan by both independent and overlapping genetic pathways in *C. elegans*. *Aging Cell* **8,** 113-127 (2009).
16. Suda, H., Sato, K. & Yanase, S. Timing mechanism and effective activation energy concerned with aging and lifespan in the long-lived and thermo sensory mutants of *Caenorhabditis elegans*. *Mech. Ageing Dev.* **133,** 600-610 (2012).
17. Schiavi, A., *et al*. Autophagy induction extends lifespan and reduces lipids content in response to frataxin silencing in *C. elegans*. *Exp. Gerontol.* **48,** 191-201 (2013).
18. Fierro-González, J. C., González-Barrios, M., Miranda-Vizuete, A. & Swoboda, P. The thioredoxin TRX-1 regulates adult lifespan extension induced by dietary restriction in *Caenorhabditis elegans*. *Biochem.* *Biophys. Res. Commun.* **406,** 478-482 (2011).
19. Cabreiro, F., *et al*. Increased life span from overexpression of superoxide dismutase in *Caenorhabditis elegans* is not caused by decreased oxidative damage. *Free Radic. Biol. Med.* **51,** 1575-1582 (2011).
20. Okuyama, T., *et al*. The ERK-MAPK pathway regulates longevity through SKN-1 and Insulin-like signaling in *Caenorhabditis elegans*. *J. Biol. Chem.* **285,** 30274-30281 (2010).
21. Iwasa, H., Yu, S., Xue, J. & Driscoll, M. Novel EGF pathway regulators modulate *C. elegans* healthspan and lifespan via EGF receptor, PLC-γ, and IP3R activation. *Aging Cell* **9,** 490-505 (2010).
22. Zhao, Y., Shao, Z. Y., Zhai, Z. W., Shen, C. & Powell-Coffman, J. A. The HIF-1 hypoxia- inducible factor modulates lifespan in *C. elegans*. *PLoS One* **4,** e6348; 10.1371/journal.pone. 0006348 (2009).
23. Van Raamsdonk, J. M. & Hekimi, S. Deletion of the mitochondrial superoxide dismutase *sod-2* extends lifespan in *Caenorhabditis elegans*. *PLoS Genet.* **5,** e1000361; 10.1371/journal. pgen.1000361 (2009).
24. Yang, W. & Hekimi, S. Two modes of mitochondrial dysfunction lead independently to lifespan extension in *Caenorhabditis elegans*. *Aging Cell* **9,** 433-447 (2010).
25. Morcos, M., *et al*. Glyoxalase-1 prevents mitochondrial protein modification and enhances lifespan in *Caenorhabditis elegans*. *Aging Cell* **7,** 260-269 (2008).
26. Shoyama, T., Ozaki, T., Ishii, N., Yokota, S. & Suda, H. Basic principle of the lifespan in the nematode *C. elegans*. *Mech. Ageing Dev.* **128,** 529-537 (2007).
27. Ghazi, A., Henis-Korenblit, S. & Kenyon, C. Regulation of *Caenorhabditis elegans* lifespan by a proteasomal E3 ligase complex. *Proc. Natl. Acad. Sci. USA* **104,** 5947-5952 (2007).
28. Ved, R., *et al*. Similar patterns of mitochondrial vulnerability and rescue induced by genetic modification of α-Synuclein, Parkin, and DJ-1 in *Caenorhabditis elegans. J. Biol. Chem.* **280,** 42655-42688 (2005).
29. Oh, S. W., *et al*. JNK regulates lifespan in *Caenorhabditis elegans* by modulating nuclear translocation of forkhead transcription factor/ DAF-16. *Proc. Natl. Acad. Sci. USA* **102,** 4494-4499 (2005).
30. Kayser, E. B., Sedensky, M. M. & Morgan, P. G. The effects of complex I function and oxidative damage on lifespan and anesthetic sensitivity in *Caenorhabditis elegans*. *Mech. Ageing Dev.* **125,** 455-464 (2004).
31. De Castro, E., de Castro, S. H. & Johnson, T. E. Isolation of long-lived mutants in *Caenorhabditis elegans* using selection for resistance to juglone. *Free Radic. Biol. Med.* **37,** 139-145 (2004).
32. Apfeld, J., O’Connor, G., McDonagh, T., Distefano, P. S. & Curtis, R. The AMP-activated protein kinase AAK-2 links energy levels and insulin-like signals to lifespan in *C. elegans*. *Genes Dev.* **18,** 3004-3009 (2004).
33. Libina, N., Berman, J. R. & Kenyon, C. Tissue-specific activities of *C. elegans* DAF-16 in the regulation of lifespan. *Cell* **115,** 489-502 (2003).
34. Meléndez, A., *et al*. Autophagy genes are essential for dauer development and life-span extension in *C. elegans*. *Science* **301,** 1387-1391 (2003).
35. Hirose, T., *et al*. Cyclic GMP-dependent protein kinase EGL-4 controls body size and lifespan in *C. elegans*. *Development* **130,** 1089-1099 (2003).
36. Tissenbaum, H. A. & Guarente, L. Increased dosage of a *sir-2* gene extends lifespan in *Caenorhabditis elegans*. *Nature* **410,** 227-230 (2001).
37. Melov, S., *et al*. Extension of life-span with superoxide dismutase/catalase mimetics. *Science* **289,** 1567-1569 (2000).
38. Bishop, N. A. & Guarente, L. Two neurons mediate diet-restriction-induced longevity in *C. elegans*. *Nature* **447,** 545-549 (2007).
39. Yen, K. & Mobbs, C. V. Dietary restriction and cold temperature both acutely reduce senescence in *C. elegans*. *Open Longevity Science* **1,** 8-13 (2007).
40. Carrano, A. C., Liu, Z., Dillin, A. & Hunter, T. A conserved ubiquitination pathway determines longevity in response to diet restriction. *Nature* **460,** 396-400 (2009).
41. Chen, D., Thomas, E. L. & Kapahi, P. HIF-1 modulates dietary restriction-mediated lifespan extension via IRE-1 in *Caenorhabditis elegans*. *PLoS Genet.* **5,** e1000486; 10.1371/journal. pgen.1000486 (2009).
42. Fieero-González, J. C., González-Barrios, M., Miranda-Vizuete, A. & Swoboda, P. The thioredoxin TRX-1 regulates adult lifespan extension induced by dietary restriction in *Caenorhabditis elegans*. *Biochem. Biophys. Res. Commun.* **406,** 478-482 (2011).
43. Lee, G. D., *et al*. Dietary deprivation extends lifespan in *Caenorhabditis elegans*. *Aging Cell* **5,** 515-524 (2006).
44. Hansen, M., *et al*. A role for autophagy in the extension of lifespan by dietary restriction in *C. elegans*. *PLoS Genet.* 4, e24; 10.1371/journal.pgen.0040024 (2008).
45. Panowski, S. H., Wolff, S., Aguilaniu, H., Durieux, J. & Dillin, A. PHA-4/Foxa mediates diet-restriction-induced longevity of *C. elegans*. *Nature* **447,** 550-555 (2007).
46. Larsen, P. L. & Clarke, C. F. Extension of life-span in *Caenorhabditis elegans* by a diet lacking coenzyme Q. *Science* **295,** 120-123 (2002).
47. Park, S. K., Link, C. D. & Johnson, T. E. Life-span extension by dietary restriction is mediated by NLP-7 signaling and coelomocyte endocytosis in *C. elegans*. *FASEB J.* **24,** 383-392 (2010).
48. So, S., Tokumaru, T., Miyahara, K. & Ohshima, Y. Control of lifespan by food bacteria, nutrient limitation and pathogenicity of food in *C. elegans*. *Mech. Ageing Dev.* **132,** 210-212 (2011).
49. Kaeberlein, T. L., *et al*. Lifespan extension in *Caenorhabditis elegans* by complete removal of food. *Aging Cell* **5,** 487-494 (2006).
50. Greer, E. L., *et al*. An AMPK-FOXO pathway mediates longevity induced by a novel method of dietary restriction in *C. elegans*. *Curr. Biol.* **17,** 1646-1656 (2007).
51. Lenaerts, I., Walker, G. A., Hoorebeke, L. V., Gems, D. & Vanfleteren, J. R. Dietary restriction of *Caenorhabditis elegans* by axenic culture reflects nutritional requirement for constituents provided by metabolically active microbes. *J. Gerontol. A Biol. Sci. Med. Sci.* **63,** 242-252 (2008).
52. Steinkraus, K. A., *et al*. Dietary restriction suppresses proteotoxicity and enhances longevity by an *hsf-1* dependent mechanism in *Caenorhabditis elegans*. *Aging Cell* **7,** 394-404 (2008).
53. Houthoofd, K., Braeckman, B., Johnson, T. E. & Vanfleteren, J. R. Life extension via dietary restriction is independent of the Ins/IGF-1 signaling pathway in *Caenorhabditis elegans*. *Exp. Gerontol.* **38,** 947-954 (2003).
54. Lakowski, B. & Hekimi, S. The genetic of caloric restriction in *Caenorhabditis elegans*. *Proc. Natl. Acad. Sci. USA* **95,** 13091-13096 (1998).
55. Schula, T. J., *et al*. Glucose restriction extends *Caenorhabditis elegans* life span by inducing mitochondrial respiration and increasing oxidative stress. *Cell Metab.* **6,** 280-293 (2007).
56. Bass, T. M., Weinkove, D., Houthoofd, K., Gems, D. & Partridge, L. Effects of resveratrol on lifespan in *Drosophila melanogaster* and *Caenorhabditis elegans*. *Mech. Ageing Dev.* **128,** 546-552 (2007).
57. Mair, W., Panowski, S. H., Shaw, R. J. & Dillin, A. Optimizing dietary restriction for genetic epistasis analysis and gene discovery in *C. elegans*. *PLoS One* **4,** e4535; 10.1371/journal. pone.0004535 (2009).
58. Smith, E. D., *et al*. Age- and calorie-independent life span extension from dietary restriction by bacterial deprivation in *Caenorhabditis elegans*. *BMC Dev. Biol.* **8,** 49; 10.1186/1471-213 x-8-49 (2008).
59. Lucanic, M., *et al*. N-acylethanolamine signalling mediates the effect of diet on lifespan in *Caenorhabditis elegans*. *Nature* **473,** 226-229 (2011).
60. Ching, T. T., Paal, A. B., Mehta, A., Zhong, L. & Hsu, A. L. *drr-2* encodes an eIF4H that acts downstream of TOR in diet-restriction-induced longevity of *C. elegans*. *Aging Cell* **9,** 545-557 (2010).
61. Sutphin, G. L. & Kaeberlein, M. Dietary restriction by bacterial deprivation increases life span in wild-derived nematodes. *Exp. Gerontol.* **43,** 130-135 (2008).
62. Mouchiroud L, *et al*. Pyruvate imbalance mediates metabolic reprogramming and mimics lifespan extension by dietary restriction in *Caenorhabditis elegans*. *Aging Cell* **10,** 39-54 (2011).
63. Tain, L. S., Lozano, E., Sáez, A. G. & Leroi, A. M. Dietary regulation of hypodermal polyploidization in *C. elegans*. *BMC Dev. Biol.* **8,** 28; 10.1186/1471-213x-8-28 (2008).
64. Lenaerts, I., van Eygen, S. & van Fleteren, J. Adult-limited dietary restriction slows Gompertzian aging in *Caenorhabditis elegans*. *Ann. N. Y. Acad. Sci.* **1100,** 442-448 (2007).
65. Schriner, S. E., *et al*. Decreased mitochondrial superoxide levels and enhanced protection against paraquat in *Drosophila melanogaster* supplemented with *Rhodiolarosea*. *Free Radic. Res.* **43,** 836-843 (2009).
66. Kang, H. L., Benzer, S. & Min, K. T. Life extension in *Drosophila* by feeding a drug. *Proc. Natl. Acad. Sci. USA* **99,** 838-843 (2002).
67. Khavinson, V. K., Izmaylov, D. M., Obukhova, L. K. & Malinin, V. V. Effect of epitalon on the lifespan increase in *Drosophila melanogaster*. *Mech. Ageing Dev.* **120,** 141-149 (2000).
68. Bauer, J. H., Goupil, S., Garber, G. B. & Helfand, S. L. An accelerated assay for the identification of lifespan-extending interventions in *Drosophila melanogaster. Proc. Natl. Acad. Sci. USA* **101,** 12980-12985 (2004).
69. Tao, D., *et al*. Trichostatin A extends lifespan of *Drosophila melanogaster* by elevating *hsp22* expression. *Acta. Biochim. Biophys. Sin.* **36,** 618-622 (2004).
70. Lee, K. S., *et al*. Curcumin extends life span, improves health span, and modulates the expression of age-associated aging genes in *Drosophila melanogaster. Rejuvenation Res.* **13,** 561-570 (2010).
71. Si, H. W., *et al*. Dietary epicatechin promotes survival of obese diabetic mice and *Drosophila melanogaster*. *J. Nutr.* **141,** 1095-1100 (2011).
72. Hada, B., *et al*. D-chiro-inositol and pinitol extend the life span of *Drosophila melanogaster. J. Gerontol. A Biol. Sci. Med. Sci.* **68,** 226-234 (2013).
73. Danilov, A., *et al*. Selective anticancer agents suppress aging in *Drosophila*. *Oncotarget* **4,** 1507-1526 (2013).
74. Xiao, F. The anti-aging effect and molecular mechanism of the major Royal Jelly proteins in *Drosophila*. *Zhejiang University* (2013).
75. Cruz, L. C., *et al*. A study on the quality and identity of Brazilian Pampa Biome Honey: evidences for its beneficial effects against oxidative stress and hyperglycemia. *Int. J. Food Sci.* **2014,** 1-11 (2014).
76. Avanesian, A., Khodayari, B., Felgner, J. S. & Jafari, M. Lamotrigine extends lifespan but compromises health span in *Drosophila melanogaster*. *Biogerontology* **11,** 45-52 (2010).
77. Bonilla, E., Medina-Leendertz, S. & Díaz, S. Extension of life span and stress resistance of *Drosophila melanogaster* by long-term supplementation with melatonin. *Exp. Gerontol.* **37,** 629-638 (2002).
78. Pan, W. G. Purified, identified of the antiaging and antioxidative compounds from the roots of *I. younghusbandii* Sprague. *Sichuan University* (2007).
79. Wen, S. P. Study on antisenescence potential of *Rosmarinus Officinalia Extract. Tianjin University of Science and Technology* (2010).
80. Navrotskaya, V. V., Oxenkrug, G., Vorobyova, L. I. & Summergrad, P. Berberine prolongs life span and stimulates locomotor activity of *Drosophila melanogaster*. *Am. J. Plant Sci.* **3,** 1037-1040 (2012).
81. Bonilla, E., *et al*. Minocycline increases the life span and motor activity and decreases lipid peroxidation in manganese treated *Drosophila melanogaster*. *Toxicology* **294,** 50-53 (2012).
82. Arcaya, J. L., Salazar, U., Morales, L., Moncada, Y. & Bonilla, E. Lower sensitivity to copper toxicity in female *Drosophila melanogaster*. *Retel* **2013,** 1-23 (2013).
83. Liu, B. J., Tian, W. Q., Zhang, Y. & Luo, J. Experiment study on the effect of Ganoderma Lucidum Triterpenoids to *Drosophila.* *Chinese Archives of Traditional Chinese Medicine* **29,** 2203-2205 (2010).
84. Hu, S. Studies on anti-aging mechanism of Phloridzin in *Drosophila Melanogaster*. *Tianjin University of Science and Technology* (2011).
85. Shao, C. Studies on anti-aging mechanism of Choerospondials Axillaries extraction in *Drosophila melanogaster*. *Tianjin University of Science and Technology* (2011).
86. Li, J. Studies on anti-aging effect of Purple Sweet Potato extraction in *Drosophila melanogaster*. *Tianjin University of Science and Technology* (2011).
87. Brack, C., Bechter-Thüring, E. & Labuhn, M. N-acetylcyteine slows down ageing and increases the life span of *Drosophila melanogaster*. *Cell Mol. Life Sci.* **53,** 960-966 (1997).
88. Peng, C., Chan, H. Y., Li, Y. M., Huang, Y. & Chen, Z. Y. Black tea theaflavins extend the lifespan of fruit flies. *Exp. Gerontol.* **44,** 773-783 (2009).
89. Peng, C., Chan, H. Y., Huang, Y., Yu, H. & Chen, Z. Y. Apple polyphenols extend the mean lifespan of *Drosophila melanogaster*. *J. Agric. Food Chem.* **59,** 2097-2106 (2011).
90. Peng, C., *et al*. Blueberry extract prolongs lifespan of *Drosophila melanogaster*. *Exp. Gerontol.* **47,** 170-178 (2012).
91. Zuo, Y., *et al*. Black rice extract extends the lifespan of fruit flies. *Food Funct.* **3,** 1271-1279 (2012).
92. Spindler, S. R., *et al*. Statin treatment increases lifespan and improves cardiac health in *Drosophila* by decreasing specific protein prenylation. *PLoS one* **7,** e39581; 10.1371/journal. pone.0039581 (2012).
93. Huangfu, J., *et al*. Anti-aging effects of astaxanthin-rich *alga Hema tococcuspluvialis* on fruit flies under oxidative stress. *J. Agric. Food Chem.* **61,** 7800-7804 (2013).
94. Zhang, Z., Han, S., Wang, H. & Wang, T. Lutein extends the lifespan of *Drosophila melanogaster*. *Arch. Gerontol. Geriatr.* **58,** 153-159 (2014).
95. Zhang, Y. X. Anti-aging and molecular mechanisms of Lotus-seed polysaccharide. *Fujian Agriculture and Forestry University* (2013).
96. Yang, X. L. The effect of *Ganoderma* *Lucidum* spore oil and the ratio of dietary sugar/protein on lifespan in *Drosophila*. *Zhejiang University* (2013).
97. Colak, D. A. The effects of *Punicagranatum L.* ethanol extract including the antioxidant flavonoids on *Drosophila melanogaster* lifespan. *J. Applied Biol. Sci.* **8,** 6-9 (2014).
98. Bahadorani, S., Bahadorani, P., Phillips, J. P. & Hilliker, A. J. The effects of vitamin supplementation on *Drosophila* life span under normoxia and under oxidative stress. *J. Gerontol. A Biol. Sci. Med. Sci.* **63,** 35-42 (2008).
99. Lavara-Culebras, E., Muňoz-Soriano, V., Gómez-Pastor, R., Matallana, E. & Paricio, N. Effects of pharmacological agents on the lifespan phenotype of *Drosophila* DJ-1β mutants. *Gene* **462,** 26-33 (2010).
100. Chen, T., *et al*. Rapamycin and other longevity-promoting compounds enhance the generation of mouse induced pluripotent sterm cells. *Aging Cell* **10,** 908-911 (2011).
101. Kim, M. S. Korean red ginseng tonic extends lifespan in *D. melanogaster*. *Biomol. Ther. (Seoul)* **21,** 241-245 (2013).
102. Lopez, T., *et al*. Green tea polyphenols extend the lifespan of male *Drosophila melanogaster* while impairing reproductive fitness. *J. Med. Food* **17,** 1314-1321 (2014).
103. Bjedov, I., *et al*. Mechanisms of life span extension by rapamycin in the fruit fly *Drosophila melanogaster*. *Cell Metab.* **11,** 35-46 (2010).
104. Sun, X., *et al*. Nutrient-dependent requirement for SOD1 in lifespan extension by protein restriction in *Drosophila melanogaster*. *Aging Cell* **11,** 783-793 (2012).
105. Chambers, R. P., *et al*. Nicotine increases lifespan and rescues olfactory and motor deficits in a *Drosophila* model of Parkinson’s disease. *Behav. Brain Res.* **253,** 95-102 (2013).
106. Kapahi, P., *et al*. Regulation of lifespan in *Drosophila* by modulation of genes in the TOR signaling pathway. *Curr. Biol.* **14,** 885-890 (2004).
107. Orr, W. C., *et al*. Overexpression of glutamate-cysteine ligase extends life span in *Drosophila melanogaster*. *J. Biol. Chem.* **280,** 37331-37338 (2005).
108. Copeland, J. M., *et al*. Extension of *Drosophila* life span by RNAi of the mitochondrial respiratory chain. *Curr. Biol.* **19,** 1591-1598 (2009).
109. Yamazaki, M., *et al*. High calorie diet augments age-associated sleep impairment in *Drosophila*. *Biochem. Biophys. Res. Commun.* **417,** 812-816 (2012).
110. Simonsen, A., *et al*. Promoting basal levels of autophagy in the nervous system enhances longevity and oxidant resistance in adult *Drosophila*. *Autophagy* **4,** 176-184 (2008).
111. Walker, D. W., Muffat, J., Rundel, C. & Benzer, S. Overexpression of a *Drosophila* homolog of apolipoprotein D leads to increased stress resistance and extended lifespan. *Curr. Biol.* **16,** 674-679 (2006).
112. Rogina, B., Reenan, R. A., Nilsen, S. P. & Helfand, S. L. Extended life-span conferred by cotransporter gene mutations in *Drosophila*. *Science* **290,** 2137-2140 (2000).
113. Libert, S., *et al*. Regulation of *Drosophila* life span by olfaction and food-derived odors. *Science* **315,** 1133-1137 (2007).
114. Burnett, C., *et al*. Absence of effects of Sir2 over-expression on lifespan in *C. elegans* and *Drosophila*. *Nature* **477,** 482-485 (2011).
115. Lee, S. H., Lee, S. K., Paik, D. & Min, K. J. Overexpression of fatty-acid-β-oxidation- related genes extends the lifespan of *Drosophila melanogaster*. *Oxid. Med. Cell Longev.* **2012,** 854502 (2012).
116. Lee, Y. N., Shim, Y. J., Kang, B. H., Park, J. J. & Min, B. H. Over-expression of human clusterin increases stress resistance and extends lifespan in *Drosophila melanogaster*. *Biochem. Biophys. Res. Commun.* **420,** 851-856 (2012).
117. Clancy, D. J., *et al*. Extension of life-span by loss of CHICO, a *Drosophila* insulin receptor substrate protein. *Science* **292,** 104-106 (2001).
118. Orr, W. C., Mockett, R. J., Benes, J. J. & Sohal, R. S. Effects of overexpression of copper-Zinc and manages superoxide dismutase, catalase, and thioredoxin reductase genes on longevity in *Drosophila melanogaster*. *J. Biol. Chem.* **287,** 26418-26422 (2003).
119. Seto, N. O., Hayashi, S. & Tener, G. M. Overexpression of Cu-Zn superoxide dismutase in *Drosophila* does not affect life-span. *Proc. Natl. Acad. Sci. USA* **87,** 4270-4274 (1990).
120. Sun, J., Folk, D., Bradley, T. J. & Tower, J. Induced overexpression of mitochondrial Mn-superoxide dismutase extends the life span of adult *Drosophila melanogaster*. *Genetics* **161,** 661-672 (2002).
121. Wang, M. C., Bohmann, D. & Jasper, H. JNK signaling confers tolerance to oxidative stress and extends lifespan in *Drosophila*. *Dev. Cell* **5,** 811-816 (2003).
122. Sanz, A., *et al*. Expression of the yeast NADH dehydrogenase Ndi1 in *Drosophila* confers increased lifespan independently of dietary restriction. *Proc. Natl. Acad. Sci. USA* **107,** 9105- 9110 (2010).
123. Mouikis, P., Hurlbut, G. D. & Artavanis-Tsakonas, S. Enigma, a mitochondrial protein affecting lifespan and oxidative stress response in *Drosophila*. *Proc. Natl. Acad. Sci. USA* **103,** 1307- 1312 (2006).
124. Bauer, J. H., Poon, P. C., Glatt-Deeley, H., Abrams, J. M. & Helfand, S. L. Neuronal expression of p53 dominant-negative proteins in adult *Drosophila melanogaster* extends life span. *Curr. Biol.* **15,** 2063-2068 (2005).
125. Wang, M. C., Bohmann, D. & Jasper, H. JNK extends life span and limits growth by antagonizing cellular and organism-wide responses to insulin signaling. *Cell* **121,** 115-125 (2005).
126. Ja, W. W., *et al*. Extension of *Drosophila* *melanogaster* life span with a GPCR peptide inhibitor. *Nat. Chem. Biol.* **3,** 415-419 (2007).
127. Muffat, J., Walker, D. W. & Benzer, S. Human ApoD, an apolipoprotein up-regulated in neurodegenerative diseases, extends lifespan and increases stress resistance in *Drosophila*. *Proc. Natl. Acad. Sci. USA* **105,** 7088-7093 (2008).
128. Runko, A. P., Griswold, A. J. & Min, K. T. Overexpression of frataxin in the mitochondria increases resistance to oxidative stress and extends lifespan in *Drosophila*. *FEBS Lett.* **582,** 715-719 (2008).
129. Fridell, Y. W, *et al*. Increased uncoupling protein (UCP) activity in *Drosophila* insulin- producing neurons attenuates insulin signaling and extends lifespan. *Aging (Albany NY)* **1,** 699-713 (2009).
130. Tricoire, H., *et al*. The steroid hormone receptor EcR finely modulates *Drosophila* lifespan during adulthood in a sex-specific manner. *Mech. Ageing Dev.* **130,** 547-552 (2009).
131. Alic, N., Hoddinott, M. P., Vinti, G. & Partridge, L. Lifespan extension by increased expression of the *Drosophila* homologue of the IGFBP7 tumour suppressor. *Aging Cell* **10,** 137-147 (2011).
132. Hoffmann, J., Romey, R., Fink, C., Yong, L. & Roeder, T. Overexpression of *Sir2* in the adult fat body is sufficient to extend lifespan of male and female *Drosophila*. *Aging (Albany NY)* **5,** 315-327 (2013).
133. Lin, Y. H., *et al*. Diacylglycerol lipase regulates lifespan and oxidative stress response by inversely modulating TOR signaling in *Drosophila* and *C. elegans. Aging Cell* **13,** 755-764 (2014).
134. Wang, D., Cui, Y., Jiang, Z. & Xie, W. Knockdown expression of eukaryotic initiation factor 5 C-terminal domain containing protein extends lifespan in *Drosophila melanogaster*. *Biochem. Biophys. Res. Commun.* **446,** 465-469 (2014).
135. Baqri, R. M., *et al*. Mitochondrial chaperone TRAP1 activates the mitochondrial UPR and extends healthspan in *Drosophila*. *Mech. Ageing Dev.* **141,** 35-45 (2014).
136. Burnett, C., *et al*. Absence of effects of Sir2 overexpression on lifespan in *C. elegans* and *Drosophila*. *Nature* **477,** 482-486 (2011).
137. Cvejic, S., Zhu, Z., Felice, S. J., Berman, Y. & Huang, X. Y. The endogenous ligand stunted of the GPCR Methuselah extends lifespan in *Drosophila*. *Nat. Cell Biol.* **6,** 540-546 (2004).
138. Chavous, D. A., Jackson, F. R. & O’Connor, C. M. Extension of the *Drosophila* lifespan by overexpression of a protein repair methyltransferase. *Proc. Natl. Acad. Sci. USA* **98,** 14814- 14818 (2001).
139. Legan, S. K., *et al*. Overexpression of glucose-6-phosphate dehydrogenase extends the life span of *Drosophila melanogaster*. *J. Biol. Chem.* **283,** 32492-32499 (2008).
140. Bross, T. G., Rogina, B. & Helfand, S. L. Behavioral, physical, and demographic changes in *Drosophila* populations through dietary restriction. *Aging Cell* **4,** 309-317 (2005).
141. Zheng, J., Mutcherson, R. & Helfand, S. L. Calorie restriction delays lipid oxidative damage in *Drosophila melanogaster*. *Aging Cell* **4,** 209-216 (2005).
142. Min, K. J. & Tatar, M. Restriction of amino acids extends lifespan in *Drosophila melanogaster*. *Mech. Ageing Dev.* **127,** 643-646 (2006).
143. Burger, J. M., Hwangbo, D. S., Corby-Harris, V. & Promislow, D. E. The functional costs and benefits of dietary restriction in *Drosophila*. *Aging Cell* **6,** 63-71 (2007).
144. Min, K. J., Flatt, T., Kulaots, I. & Tatar, M. Counting calories in *Drosophila* diet restriction. *Exp. Gerontol.* **42,** 247-251 (2007).
145. Che, N. K. The effect of dietary restriction on the lifespan and reproduction of male *Drosophila melanogaster*. *Shanxi Normal University* (2007).
146. Dick, K. B., Ross, C. R. & Yampolsky, L. Y. Genetic variation of dietary restriction and the effects of nutrient-free water and amino acid supplements on lifespan and fecundity of *Drosophila*. *Genet. Res.* **93,** 265-273 (2011).
147. Zeng, C., *et al*. Gender-specific prandial response to dietary restriction and oxidative stress in *Drosophila melanogaster*. *Fly* **5,** 174-180 (2011).
148. Sun, X., *et al*. Nutrient-dependent requirement for SOD1 in lifespan extension by protein restriction in *Drosophila melanogaster*. *Aging Cell* **11,** 783-793 (2012).
149. Ja, W. W., *et al*. Water- and nutrient-dependent effects of dietary restriction on *Drosophila* lifespan. *Proc. Natl. Acad. Sci. USA* **106,** 18633-18637 (2009).
150. Pletcher, S. D., *et al*. Genome-wide transcript profiles in aging and calorically restricted *Drosophila melanogaster*. *Curr. Biol.* **12,** 712-723 (2002).
151. Magwere, T., Chapman, T. & Partridge, L. Sex differences in the effect of dietary restriction on lifespan and mortality rates in female and male *Drosophila melanogaster*. *J. Gerontol. A Biol. Sci. Med. Sci.* **59,** 3-9 (2004).
152. Mair, W., Piper, M. D. & Partridge, L. Calories do not explain extension of life span by dietary restriction in *Drosophila*. *PLoS Biol.* **3,** e223; 10.1371/journal.pbio.0030223 (2005).
153. Grandison, R. C., Piper, M. D. & Partridge, L. Amino-acid imbalance explains extension of lifespan by dietary restriction in *Drosophila*. *Nature* **462,** 1061-1065 (2009).
154. Kabil, H., Kabil, O., Banerjee, R., Harshman, L. G. & Pletcher, S. D. Increased transsulfuration mediates longevity and dietary restriction in *Drosophila*. *Proc. Natl. Acad. Sci. USA* **108,** 16831-16836 (2011).
155. Troen, A. M., *et al*. Lifespan modification by glucose and methionine in *Drosophila melanogaster* fed a chemically defined diet. *Age* **29,** 29-39 (2007).
156. Tu, M. P. & Tatar, M. Juvenile diet restriction and the aging and reproduction of adult *Drosophila melanogaster*. *Aging Cell* **2,** 327-333 (2003).
157. Soh, J. W., Hotic, S. & Arking, R. Dietary restriction in *Drosophila* is dependent on mitochondrial efficiency and constrained by pre-existing extended longevity. *Mech. Ageing Dev.* **128,** 581-593 (2007).
158. Vigne, P. & Frelin, C. Diet dependent longevity and hypoxic tolerance of adult *Drosophila melanogaster*. *Mech. Ageing Dev.* **128,** 401-406 (2007).
159. Metaxakis, A. & Partridge, L. Dietary restriction extends lifespan in wild-derived populations of *Drosophila melanogaster*. *PLoS One* **8,** e74681; 10.1371/journal.pone.0074681 (2013).
160. Emran, S., Yang, M., He, X., Zandveld, J. & Piper, M. D. Target of rapamycin signaling mediates the lifespan-extending effects of dietary restriction by essential amino acid alteration. *Aging (Albany NY)* **6,** 390-398 (2014).
161. Lee, B. C., *et al*. Methionine restriction extends lifespan of *Drosophila melanogaster* under conditions of low amino-acid status. *Nat. Commun.* **5,** 3592 (2014).

**Supplementary File S2. References of articles from which the survival curves were extracted to assess the stability of our methods.**

1. Büchter, C., *et al*. TSG (2,3,5,4’-Tetrahydroxystilbene-2-O-β-D-glucoside) from the Chinese Herb *Polygonum multiflorum* increases life span and stress resistance of *Caenorhabditis elegans*. *Oxid. Med. Cell Longev.* **2015,** 124357; 10.1155/2015/124357 (2015).
2. Viswanathan, M., Kim, S. K., Berdichevsky, A. & Guarente, L. A role for SIR-2.1 regulation of ER stress response genes in determining *C. elegans* life span. *Dev. Cell* **9,** 605-615 (2005).
3. Ye, K., *et al*. Resveratrol attenuates radiation damage in *Caenorhabditis elegans* by preventing oxidative stress. *J. Radiat. Res.* **51,** 473-479 (2010).
4. Greer, E. L. & Brunet, A. Different dietary restriction regimens extend lifespan by both independent and overlapping genetic pathways in *C. elegans*. *Aging Cell* **8,** 113-127 (2009).
5. Upadhyay, A., Chompoo, J., Taira, N., Fukuta, M. & Tawata, S. Significant longevity- extending effects of *Alpinia zerumbet* leaf extract on the life span of *Caenorhabditis elegans. Biosci. Biotechnol. Biochem.* **77,** 217-223 (2013).
6. Morselli, E., *et al*. Caloric restriction and resveratrol promote longevity through the Sirtuin-1- dependent induction of autophagy. *Cell Death Dis.* **1,** e10; 10.1038/cddis.2009.8 (2010).
7. Chen, W., Rezaizadehnajafi, L. & Wink, M. Influence of resveratrol on oxidative stress resistance and life span in *Caenorhabditis elegans*. *J. Pharm. Pharmacol.* **64,** 682-688 (2013).
8. Kashyap, S. S., *et al*. *Caenorhabditis elegans* dnj-14, the orthologue of the DNAJC5 gene mutated in adult onset neuronal ceroidlipofuscinosis, provides a new platform for neuroprotective drug screening and identifies a SIR-2.1 independent action of resveratrol. *Hum. Mol. Genet.* **23,** 5916-5927 (2014).
9. Gruber, J., Tang, S. Y. & Halliwell, B. Evidence for a trade-off between survival and fitness caused by resveratrol treatment of *Caenorhabditis elegans. Ann. N. Y. Acad. Sci.* **1100,** 530- 542 (2007).
10. Wood, J. G., *et al*. Sirtuin activators mimic caloric restriction and delay ageing in metazoans. *Nature* **430,** 686-689 (2004).
